# Functions of Calcium-Dependent Protein Kinases in Plant Innate Immunity

**DOI:** 10.3390/plants3010160

**Published:** 2014-03-05

**Authors:** Xiquan Gao, Kevin L. Cox, Ping He

**Affiliations:** 1 State Key Laboratory of Crop Genetics and Germplasm Enhancement, College of Agriculture, Nanjing Agricultural University, Nanjing 210095, China; 2 Department of Plant Pathology and Microbiology, Institute for Plant Genomics and Biotechnology, Texas A&M University, College Station, TX 77843, USA; 3 Department of Biochemistry and Biophysics, Institute for Plant Genomics and Biotechnology, Texas A&M University, College Station, TX 77843, USA

**Keywords:** calcium-dependent protein kinase, PAMP-triggered immunity, effector-triggered immunity, phosphorylation

## Abstract

An increase of cytosolic Ca^2+^ is generated by diverse physiological stimuli and stresses, including pathogen attack. Plants have evolved two branches of the immune system to defend against pathogen infections. The primary innate immune response is triggered by the detection of evolutionarily conserved pathogen-associated molecular pattern (PAMP), which is called PAMP-triggered immunity (PTI). The second branch of plant innate immunity is triggered by the recognition of specific pathogen effector proteins and known as effector-triggered immunity (ETI). Calcium (Ca^2+^) signaling is essential in both plant PTI and ETI responses. Calcium-dependent protein kinases (CDPKs) have emerged as important Ca^2+^ sensor proteins in transducing differential Ca^2+^ signatures, triggered by PAMPs or effectors and activating complex downstream responses. CDPKs directly transmit calcium signals by calcium binding to the elongation factor (EF)-hand domain at the *C*-terminus and substrate phosphorylation by the catalytic kinase domain at the *N*-terminus. Emerging evidence suggests that specific and overlapping CDPKs phosphorylate distinct substrates in PTI and ETI to regulate diverse plant immune responses, including production of reactive oxygen species, transcriptional reprogramming of immune genes, and the hypersensitive response.

## 1. Introduction

Calcium ions (Ca^2+^) are one of the most important cellular ionic species that function as second messengers in many aspects of biological processes in plants and animals. When the host plants encounter a diverse number of physiological stimuli or stresses, including cold, drought, salinity stresses, and pathogen or herbivory attacks, an increase in cytosolic free Ca^2+^ levels ((Ca^2+^)cyt) is rapidly triggered [1]. The Ca^2+^ signals are subsequently sensed and translated into intracellular responses, mainly via different Ca^2+^ sensor proteins, some of which are highly conserved in all eukaryotic organisms, thereby triggering complex downstream signaling in response to developmental and environmental cues. The temporal and spatial changes of Ca^2+^ binding to the sensor proteins are monitored by the conformational changes of sensor proteins in a Ca^2+^-dependent manner [2,3,4].

## 2. Calcium and Calcium Sensor Proteins

There are two types of Ca^2+^ sensor proteins, one of which relays calcium signals, such as calcineurin B-like/CBL-interacting protein kinases (CBL/CIPKs) and calmodulin proteins (CaMs), while the other constitute sensor protein kinases, such as calcium-dependent protein kinases (CDPKs) and calmodulin-dependent protein kinases (CaMKs) [5,6]. CaMs are found ubiquitously in all eukaryotes, generally being defined as CaM, CaM-like (CML), and CaM-related proteins; all of which typically contain four elongation factor (EF)-hand domains for calcium binding [7,8,9]. EF-hands are found in pairs in calcium sensor proteins; each of which is considered the basic functional unit to stabilize the protein and facilitate high-affinity binding to Ca^2+^ [9]. The binding of calcium to the EF-hands subsequently leads to the conformational change of the CaM globular structure, which allows the interaction of CaMs with their target proteins [10,11]. Multiple target proteins have been identified and the interaction of CaM/CML with the target proteins is proposed to function in diverse cellular responses, including transcriptional reprogramming, activation of the phosphorylation cascade, and accumulation of secondary metabolites [1,12,13]. For example, CaMs/CMLs interact with no pollen germination 1 (NPG1) [14], the kinesin-like calmodulin binding protein (KCBP) [15], and the *Arabidopsis thaliana* Na^+^/H^+^ antiporter 1 (AtNHX1) [16] to modulate development and growth processes. CaM/CMLs also target the barley recessive resistance protein MLO [17], *Arabidopsis thaliana* cyclic nucleotide-gated ion channel 2 (AtCNGC2) [18], nicotinamide adenine dinucleotide (NAD^+^) kinases (NADKs) [19], the NAC and WRKY family of transcription factors [20,21], and *Arabidopsis* calmodulin binding protein *60* (CBP60) [22] to regulate defense responses to pathogen attack.

Although CBLs contain three EF-hand motifs, unlike CaMs, CBLs were only found in plants and some protists [2]. Substitutions within the Ca^2+^-binding loops of CBLs indicate that the Ca^2+^-binding activity of CBLs is different from the EF-hands of CaM, and different CBLs display differential Ca^2+^ affinities to Ca^2+^, supporting the complexity and flexibility of CBLs in sensing Ca^2+^ signals [2]. CBL-interacting protein kinases (CIPKs), the direct interacting substrate proteins of CBLs, belong to a type of kinase that contain domains related to the sucrose non-fermenting-like kinase 1 (SNF1) and AMP-dependent kinases (AMPKs) from yeast and mammals, respectively [23]. CBLs interact with CIPKs via the conserved NAF domain at the CIPK *C*-terminus, subsequently releasing the kinase from auto-inhibition [24]. It seems that the competitive recruitment of different CIPKs by various CBLs, and *vice versa*, at distinct subcellular compartments dictates not only the specificity, but also the versatility of the CBLs/CIPKs complex in decoding Ca^2+^ signals, thereby leading to subsequent response outputs [1,23].

## 3. Structure, Distribution, and Physiological Functions of CDPKs

Distinctly different from CaM and CBLs, which do not exert their function via possessing any intrinsic activity, but transduce calcium signals to target proteins, CDPKs and CaMKs can directly transmit the calcium signals by calcium binding to the EF-hands at their *C*-terminus and by substrate phosphorylation via the catalytic kinase domain at their *N*-terminus. Upstream of the kinase domain, there is a variable *N*-terminal domain ranging in length from 40–180 amino acids that has very low homology among different family members [5]. Many CDPKs contain potential myristoylation and palmitoylation sites at the beginning of their highly variable *N*-terminal domains, implicating the association of CDPKs with the plasma membrane. In addition to plasma membrane localization, CDPKs are also proposed to be localized in other subcellular organelles, including the cytosol, endoplasmic reticulum, and peroxisomes [6,25,26,27]. Furthermore, a conserved PEST (proline, glutamine, serine and threonine) domain, which exerts rapid proteolytic degradation, has also been found in the *N*-terminus of some CDPKs [25]. The conserved protein kinase domain contains serine/threonine residues and the activation loop has acidic residues to prevent the loop from being unnecessarily phosphorylated [5,28,29]. The protein kinase domain links to a calmodulin-like domain through a pseudosubstrate-containing auto-inhibitory junction domain, which is capable of inhibiting kinase activity via interaction with the active site in the absence of Ca^2+^ and keeping CDPKs in a state of low activity [28,29]. Upon calcium influx into the cell, the intramolecular interaction between the CaM-like domain and the auto-inhibitory domain causes the conformational change of the protein, and leads to the activation of the kinase domain [28,29,30,31,32,33].

CDPKs have been found widely in flowering plants, algae, and certain apicomplexa protists [30]. The *Arabidopsis* and rice genomes encode a large gene family with 34 and 29 members, respectively [6,34]. The genome-wide analysis of *Arabidopsis* revealed the occurance of gene duplication and subsequent divergence of CDPKs with distinct functions. The large gene family of CDPKs consist of at least 12 subfamilies that are proposed to be derived from a common ancestor of monocots and eudicots [28]. In addition to the model plants *Arabidopsis* and rice, the diversity in the multigene family of CDPKs also exists in other plant species, including soybean *(Glycine max*), tomato (*Lycopersicon esculentum*), and maize (*Zea mays*) [6,28].

In plants, CDPKs and related kinases (CRKs) have been reported to function in various developmental processes [35], including pollen tube growth [36], root development [37], stem elongation and vascular development [38], as well as cell division and differentiation [5]. Moreover, CDPKs are also involved in plant responses to diverse abiotic stresses. For instance, transient expression of *Arabidopsis thaliana* calcium-dependent protein kinase 10 (AtCPK10) or AtCPK30 could strongly activate the expression of a stress-regulated promoter that responds to multiple stresses, including cold, drought, and salt [27]. Accumulating evidence suggests that plant CDPKs are also involved in phytohormone-mediated signaling pathways. Using a combination of genetic and biochemical approaches, it has been shown that *Arabidopsis* CPK4 and CPK11 could phosphorylate abscisic acid (ABA)-responsive element-binding factor 1 (ABF1) and ABF4, to regulate ABA signaling during seed germination, seedling growth, guard cell opening, and the insensitivity to salt stress [39]. A recent study showed that *Arabidopsis* CPK6 positively regulated the activation of ion channels in ABA signaling, and activated the stomatal closure by yeast elicitor (YEL) in *Arabidopsis* [40]. Mutation of *Arabidopsis* CPK6 impaired the methyl jasmonate (MeJA)-induced stomatal closure, implying that CPK6 also functions as a positive regulator in MeJA signaling in *Arabidopsis* guard cells [40,41].

## 4. Two Branches of Plant Innate Immunity

**Figure 1:**
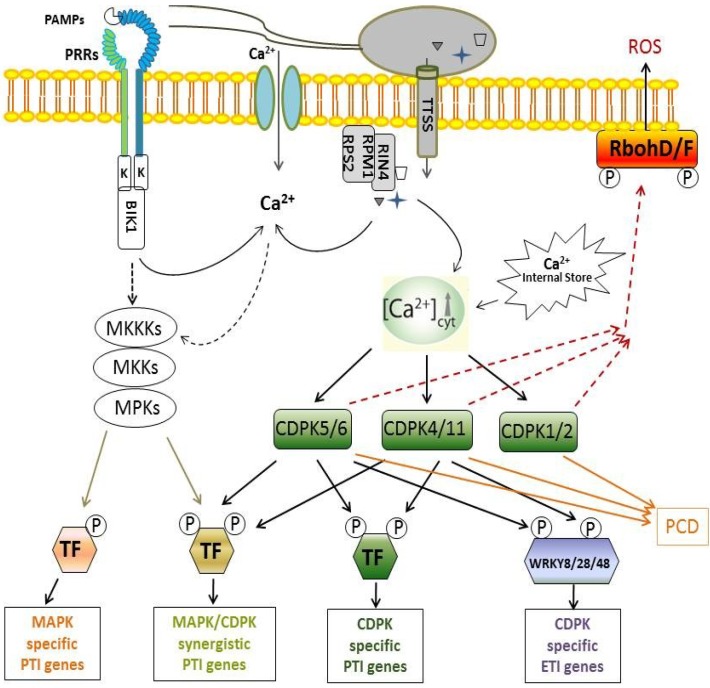
Model of calcium-dependent protein kinase (CDPK)-mediated plant innate immune signaling. Plants employ cell surface pattern-recognition receptors (PRRs) or intracellular nucleotide-binding domain leucine-rich repeat (NB-LRR or plant NLR) proteins to perceive pathogen-associated molecular patterns (PAMPs) or effector proteins, respectively. The signatures of increased cytosolic calcium (Ca^2+^) levels triggered by interaction of PAMP-PRR or effector-NLR are sensed by specific CDPKs, which trigger subsequent activation of distinct immune events. CDPKs could act synergistically or independently with the mitogen-activated protein kinase (MAPK) cascade, which consititutes three tiered kinases, MAPK (MPK), MAPK kinase (MKK) and MKK kinase (MKKK), in regulating reactive oxygen species (ROS) production and transcriptional reprogramming in PAMP-triggered immunity (PTI) signaling. On the other hand, CDPKs phosphorylate distinct substrates in regulating bifurcate effector-triggered immunity (ETI) signaling, including phosphorylation of WRKY8/28/48 transcription factors (TF) for immune gene expression and respiratory burst oxidase homolog D/F (RbohD/F) for ROS production.

Similar to animals, plants possess a surveillance system mediated by pattern-recognition receptors (PRRs) to sense invading microorganisms and trigger potent immune responses via detection of conserved pathogen or microbe-associated molecular patterns (PAMPs/MAMPs) (Figure 1) [42,43]. Plants utilize receptor-like kinases (RLKs) localized on the cell surface to perceive PAMPs, thereby activating PAMP-triggered immunity (PTI) [44,45]. The well-studied PAMPs include flg22 derived from the bacterial flagellin and elf18 from the elongation factor Tu (EF-Tu), peptidoglycan (PGN) [46,47], lipopolysaccharides (LPS) [48], as well as chitin from fungal cell wall polymers [49]. Different PAMPs likely trigger distinct, yet convergent, downstream signaling pathways. For instance, perception of flg22 by the PRR protein flagellin sensing 2 (FLS2) in *Arabidopsis* involves a complex formation of FLS2 with the brassinosteroid (BR)-insensitive 1 (BRI1)-associated receptor kinase 1(BAK1) [50], which appears to be a shared component of the signaling complex activated by multiple PAMPs. A cytoplasmic receptor kinase *Botrytis*-induced kinase 1 (BIK1) was found to constitutively associate with FLS2 and BAK1. Phosphorylation of BIK1 by BAK1 results in the activation of downstream PTI signaling, whereas phosphorylation of BIK1 by BRI1 negatively regulates plant hormone BR signaling [51,52,53]. Moreover, two E3-ubiquitin ligases, PUB12 and PUB13, function as negative regulators via BAK1 phosphorylation-mediated interaction and polyubiquitination of FLS2, which leads to the degradation of FLS2 to down-regulate flagellin signaling [54]. 

To encounter PTI, successful pathogens often secrete various virulence proteins, so-called effectors, through the bacterial type III secretion system or fungal haustorium. The effectors are in turn recognized by a type of intracellular nucleotide-binding domain leucine-rich repeat (NB-LRR or plant NLR)proteins [55,56,57], structurally similar to mammalian nucleotide-binding oligomerization domain receptors (NOD-like receptor or NLR) that sense intracellular PAMPs [58]. Recognition of effectors by plant NLRs eventually initiates effector-triggered immunity (ETI) [45,59,60,61]. Plant NLRs are also known as resistance (R) proteins, which have been widely used in agriculture for the improvement of resistance to a wide range of pathogens [62,63]. Plant NLR proteins often trigger diverse immune responses, including apoptosis-like programmed cell death (PCD), also called hypersensitive response (HR), to restrict the growth and spread of biotrophic pathogens, production of reactive oxygen species (ROS), as well as transcriptional reprogramming of immune genes [61,64]. The *Arabidopsis* NLR protein resistance to *Pseuodomonas syringae* 2 (RPS2) initiates the resistance to *Pseudomonas syringae* carrying AvrRpt2, whereas resistance to *Pseuodomonas syringae pv. maculicola* 1 (RPM1) recognizes two unrelated *P. syringae* effectors: AvrRpm1 and AvrB [65,66,67]. AvrRpt2, AvrRpm1, and AvrB activate ETI signaling by modification of host target RPM1-interacting 4 (RIN4), which is sensed by the corresponding RPS2 and RPM1 proteins [68]. RPM1 has been reported to localize and function on the plasma membrane [69]. It is likely that different NLR proteins deploy distinct mechanisms through recognizing and interacting with the effectors and/or downstream components in multiple subcellular compartments to trigger complex downstream immune responses. 

Ca^2+^ influx is one of the earliest responses upon recognition of pathogens in plant innate immunity, and distinct calcium signatures have been observed in plant PTI and ETI signaling. For example, different PAMPs or elicitors, such as flg22 and elf18, triggered the rapid and transient increase of cytosolic Ca^2+^ concentration in distinct cellular compartments, including the cytosol, nucleus, mitochondria, or chloroplasts [70]. While challenging of plants with bacteria carrying *avrRpm1* or *avrB* induced a much prolonged and sustained increase of cytosolic Ca^2+^ in *Arabidopsis* [71], calcium-channel blockers completely suppressed AvrRpm1-meditated cell death. The data suggest that Ca^2+^ signaling plays essential roles in modulating diverse aspects of innate immunity through differential Ca^2+^ sensory machineries [18,71].

## 5. Functions of CDPKs in PTI Signaling

### 5.1. CDPK-Regulated Transcriptional Reprogramming in PTI

Accumulating evidence has suggested the essential roles of CDPKs in plant basal resistance mediated by PTI signaling. For instance, *Triticum aestivum* calcium-dependent protein kinase 2 (TaCDPK2), a wheat CDPK, was found to be required for powdery mildew resistance, and the overexpression of TaCDPK2 in rice resulted in an enhanced resistance to bacterial blight (*Xanthomonas oryzae pv. oryzae*, Xoo) [72]. In *Arabidopsis*, overexpression of AtCPK1 confers broad-spectrum resistance to both bacteria and fungi [73]. CDPKs function in innate immunity by controlling transcriptional reprogramming of immune genes. In *Arabidopsis*, for example, a functional genomic screen using a flg22-responsive reporter gene, *NHL10-LUC* (*NDR1/HIN1-like10-luciferase*), as a marker enabled the identification of a group of closely related CDPKs, AtCPK4, AtCPK5, AtCPK6 and AtCPK11, as early transcriptional regulators in flg22 signaling [74]. While the Ca^2+^ channel blockers La^3+^ and Gd^3+^ eliminated the activation of *NHL10-LUC* by flg22, active AtCPK4, AtCPK5, AtCPK6 and AtCPK11 induced *NHL10-LUC* to a level comparable to that by flg22. Moreover, three genes (*PROPEP1*, *PROPEP2,* and *PROPEP3*) encoding endogenous defense peptides were also quickly activated by flg22 and active AtCPK5 and AtCPK11. More importantly, ectopic expression of active AtCPK5 or AtCPK11 induced a set of largely overlapping genes with flg22 and other PAMP-induced early genes [74], suggesting that CDPKs are the convergent point of PTI signaling triggered by diverse PAMPs. Similarly, transient expression of constitutively active *Nicotiana benthamiana* calcium-dependent protein kinase 2 (NtCDPK2) triggered the accumulation of jasmonic acid (JA) and ethylene (ET), but not salicyclic acid (SA), accompanied by the activation of JA- and ET-regulated genes [75], indicating the complexity of CDPKs in regulating innate immunity and defense-related phytohormone signaling. Future identification of transcription factors that function downstream of CDPKs will enable the elucidation of how CDPKs regulate the transcriptional reprogramming of defense genes in PTI signaling.

### 5.2. CDPK-Regulated ROS Production in PTI

Two potato CDPKs, *Solanum tuberosum* calcium-dependent protein kinase 4 (StCDPK4) and StCDPK5, were reported to directly phosphorylate *Solanum tuberosum* respiratory burst oxidase homologue B (StRbohB) to activate ROS production. The *N*-terminal variable domains seem to be required for phosphorylation of StRbohB and ROS production, as mutations of *N*-terminal myristoylation and palmitoylation sites in the V-domain resulted in the incapability of StCDPK5 to phosphorylate StRbohB [76]. In line with these findings, a recent report also showed that activation of *Arabidopsis* AtCPK5 resulted in enhanced SA-mediated resistance to *Pseudomonas syringae* pv. *tomato* strain DC3000 (*Pst* DC3000), defense gene expression, and ROS production via direct phosphorylation of RbohD, further confirming the potential functions of CDPKs in PTI signaling via ROS production [77]. The phosphopeptide analysis, based on a targeted mass spectrometry (MS) approach, selected reaction monitoring (SRM), supported that AtCPK5-regulated ROS production is flg22 ligand-dependent [77]. On the other hand, rice CDPK12 seems to play a negative role in ROS production as well as the immunity to both virulent and avirulent blast fungus [78]. The complexity of CDPKs in regulating ROS production may be mediated by differential phosphorylation of Rboh family members.

Although it has been suggested that substrate itself, Ca^2+^ affinities of CDPKs, the kinetic parameters of certain substrate and the phosphorylation sites contribute to the specificity, how CDPKs determine specific substrates remains enigmatic [2,79]. It has been proposed that substrate, specificity of kinases, largely relies on the interaction between the activation domain of kinases and the sequences surrounding the phosphorylation sites of substrates [26]. Until now, multiple phosphorylation motifs of CDPKs have been identified through in-depth analysis, including basic-XX-S/T and S-X-basic, which have been extensively and effectively used to search plant proteomes for predicting potential CDPK substrates [30]. For instance, StCDPKs were found to phosphorylate StRbohB at residues Serine (Ser)-82 and Ser-97 [76], while AtCPK5 showed strong phosphorylation activity on AtRbohD at Ser-39, Ser-148, Ser-163, and Ser-347 [77].

### 5.3. Crosstalk of MAPKs and CDPKs

The mitogen-activated protein kinase (MAPK) cascade is a prevalent component in PTI signaling [61]. While CDPKs have been identified at different cellular compartments [6,25,26,27], MAPKs were reported to be mainly localized in the cytosol and the nucleus [80]. Despite this, crosstalk between MAPKs and CDPKs has been implicated in plant innate immune responses. For instance, both MAPKs and CDPKs could regulate 1-aminocyclopropane-1-carboxylic acid synthase (ACS) activity, likely through phosphorylation by both MAPKs and CDPKs [81]. Moreover, CDPK signaling compromised stress-induced MAPK activation in tobacco, depending on ethylene synthesis and perception [75]. In addition, global gene expression analysis revealed that CDPKs and MAPKs function either synergistically or independently in PTI responses. In this study, different sets of flg22-inducible genes in protoplasts expressing the active form of AtCPK5 and AtMKK4 were identified to be either MAPK specific (*FRK1*), MAPK dominant (*CYP81F2*), CDPK specific (*PHI-1*), or CDPK/MAPK synergistic (*NHL10*) [74]. However, overexpression of active AtCPK5 in mesophyll protoplasts did not activate AtMPK3 and AtMPK6 [74]. This data indicates that a concerted activation of both CDPK and MAPK pathways controls the specific yet overlapping downstream immune signaling events in PTI (Figure 1).

## 6. Functions of CDPKs in ETI Signaling

### 6.1. CDPK-Regulated Transcriptional Reprogramming in ETI

So far, there are limited data showing that CDPKs regulate immune gene expression in ETI signaling. A previous study showed that overexpression of AtCPK1 resulted in an increase of SA levels through the activation of SA biosynthesis and regulatory genes, such as phytoalexin-deficient 4 (*PAD4*) and SA induction deficient 2/isochorismate synthase 1 (*SID2/ICS1*) [73]. Using a functional genomic screen, in combination with a genetic study, we found recently that constitutive expression of active form of AtCPK4, AtCPK5, AtCPK6, or AtCPK11 moderately inducedthe expression of the ETI marker gene *WRKY46*. This activation could be synergistically enhanced with the co-expression of a group of specific WRKY transcription factors, including WRKY8, WRKY28, and WRKY48, from subgroup IIc in *Arabidopsis*. Supporting this, bioinformatic analyses suggested that the amino acid sequence surrounding Threonine 247 (T247) and T248 of WRKY48 [basic-X-TT-X-X-X-X-hydrophobic (h)-basic] closely matches an optimal phosphorylation substrate target of CDPKs (basic-h-X-basic-X-XS/T-X-X-X-h-basic) [6,82], indicating that CDPKs control the transcriptional reprogramming likely through phosphorylating WRKYs in ETI signaling. Indeed, the experimental *in vitro* phosphorylation assay demonstrated that AtCPK4, AtCPK5, AtCPK6, or AtCPK11 strongly phosphorylated WRKY8, WRKY28, and WRKY48, resulting in enhanced binding of WRKYs to the cis-element in the promoter region of immune responsive gene *WRKY46* [82]. Future study will focus on the identification of CDPK-mediated WRKY targeted genes and elucidation their importance in ETI signaling.

### 6.2. CDPK-Regulated ROS Production in ETI

PAMP-induced ROS production occurs immediately, usually between 2–30 min upon PAMP elicitation or pathogen attack [74]. In contrast to this rapid and transient ROS burst in PTI signaling, effector-induced ROS production seems to be more prolonged and robust [71], which is correlated with the sustained increase of Ca^2+^ levels upon elicitation in effector-NLR interaction. The involvement of CDPKs in the production of ROS in ETI signaling was supported by *Arabidopsis cpk1/2* mutants showing reduced ROS accumulation on the leaves challenged with *Pst* DC3000 carrying *avrRmp1* or *avrRpt2* with a diaminobenzidine (DAB) staining assay. An immunocomplex kinase assay further demonstrated that AtCPK1 and AtCPK2, as well as AtCPK4 and AtCPK11, strongly phosphorylated AtRbohD and AtRbohF which are responsible for the NLR-triggered ROS production [82]. While StCDPKs phosphorylated StRbohB at residues Ser-82 and Ser-97 [76], mutation of Ser-148 in AtRbohD which corresponds to Ser-97 in StRbohB, but not Ser-133 in AtRbohD which corresponds to Ser-82 in StRbohB, resulted in the reduced phosphorylation of AtRbohD by AtCPK2, AtCPK4, and AtCPK11 [82]. Comparing to AtCPK1, AtCPK5 and AtCPK6 only weakly phosphorylated AtRbohD and AtRbohF, suggesting the specificity of CDPKs in phosphorylating Rbohs. A recent report also suggested that AtCPK5 phosphorylated AtRbohD with the peptide of AtRbohD amino acid 143–152 as a substrate and full length AtCPK5 protein as a kinase and this phosphorylation associated with ROS production in PTI signaling [76]. Together, these findings suggest the specificity and diversity of CDPK substrate phosphorylation in regulating ROS production in both PTI and ETI signaling pathways.

### 6.3. CDPK-Regulated HR in ETI

Distinct from the rapid and transient increase of cytosolic Ca^2+^ concentration induced by PAMPs [83,84,85], inoculation with bacteria carrying *avrRpm1*, *avrB* or *avrRpt2* triggered a prolonged and sustained increase of cytosolic Ca^2+^ concentration, which is associated with PCD in *Arabidopsis* leaves [71,86]. Furthermore, expression of AvrRpm1, AvrB, or AvrRpt2 in *Arabidopsis* protoplasts triggered distinct kinetics of PCD as detected by Evan’s blue staining with an earlier PCD occurring as early as 2 hours post-transfection (hpt) for AvrRpm1 or AvrB, and a later PCD observed at 16 hpt for AvrRpt2 [82]. The specificity and redundancy of individual CDPKs that control PCD in ETI signaling have been demonstrated recently. *Arabidopsis* CPK1, CPK2, CPK5, CPK6 and probably CPK4 and CPK11 function synergistically in controlling PCD, as the *cpk1/2/5/6* quadruple mutants, but not double mutants, displayed a reduced HR [82]. In line with this finding, silencing of NtCDPK2 subfamily resulted in the compromised induction of Avr9/Cf-9-dependent HR-like PCD symptom in *Nicotiana benthamiana* [87], while transgenic potato plants carrying *StCDPK5* showed enhanced HR-like PCD and resistance to the hemibiotrophic pathogen *Phytophthora infestans* [76]. In addition, transient expression of a constitutively active form of barley *Hordeum vulgare* calcium-dependent protein kinase 4 (HvCDPK4) in *Nicotiana benthamiana* triggered cell death in tobacco mesophyll cells [88], whereas virus-induced gene silencing (VIGS) of the *NtCDPK2* and *NtCDPK3* gene family eliminated the HR elicited by race-specific pathogens [87]. Identification of CDPK substrates that control PCD will shed light on how CDPKs regulate PCD in ETI signaling. 

### 6.4. Nuclear Dynamics of CDPKs in Immune Signaling

Although it has been widely known that CDPKs contain myristoylation and palmitoylation sites in the *N*-terminal variable domain indicating their potential plasma membrane localization, it has been suggested that a second post-translational modification, such as phosphorylation, affects the translocation of CDPKs from the membranes to other organelles, such as the cytosol or the nucleus, upon activation [89,90]. In line with this hypothesis, the ice plant (*Mesembryanthemum crystallinum*) McCPK1 and the groundnut (*Arachis hypogaea*) AhCPK2 were reported to accumulate largely in the nucleus upon environmental stress [90,91]. A similar study showed that calcium/calmodulin-dependent protein kinase (CCaMK) interacts with and phosphorylates the nuclear localized coiled-coil protein CYCLOPS required for both arbuscular mycorrhizal (AM) and rhizobial infection [92,93].We also showed that a significant portion of AtCPK5 re-localized to the nucleus where it could interact and phosphorylate WRKY8, WRKY28 and WRKY48 upon eliciting the *Arabidopsis* cells with effector protein AvrRpt2 [82]. Even though that several transcription factors, including WRKYs, REPRESSION OF SHOOT GROWTH (RSG), and ABF4 were identified as CDPK substrates [82,94], it is tempting to speculate that the translocation of CDPKs to the nucleus upon elicitation by effectors is essential to relay downstream signaling in ETI. Previous reports showed that several R proteins required effector-induced nuclear translocation for full downstream immune signaling [95,96,97]. Using the recombinant effector protein AvrRpt2 tagged with Nuclear Export Sequence (NES) or the mutated sequence of NES (nes) at its *C*-terminus, we found that AvrRpt2-NES did not reduce the ability to activate *WRKY46::LUC* compared to AvrRpt2 or AvrRpt2-nes [82,98]. More importantly, AtCPK5-NES was no longer able to display synergistic effect with WRKY8 or WRKY48 to activate *WRKY46::LUC* compared to AtCPK5 itself. Therefore, these data together demonstrated that the nuclear localization and subcellular dynamics of CDPKs are essential to orchestrating bifurcate ETI signaling via distinct substrate specificity and subcellular dynamics, for instance, to interact with and phosphorylate transcription factors in the nucleus to regulate transcriptional reprogramming crucial for restriction of pathogen growth. 

## 7. Conclusions and Perspectives

Extensive research efforts made in the past decade using integrative approaches have significantly advanced our understanding of how plant CDPKs function as the versatile Ca^2+^-sensor proteins in translating Ca^2+^ signatures into downstream responses, such as phosphorylation events and transcriptional reprogramming of immune genes upon pathogen challenges.With the unique binding motif EF-hands, as well as the catalytic kinase domain at the *N*-terminus distinct from other Ca^2+^-sensor proteins, such as CaMs, CMLs, and CBL/CIPK complexes, CDPKs exert their functions via not only direct calcium ion binding but also through the phosphorylation of their substrate proteins. Interaction of CDPKs with their target proteins or substrates consecutively leads to the regulation of diverse immune responses, including ROS production and regulation of immune gene expression in both PTI and ETI, as well as HR-like PCD in ETI (Figure 1). Apparently, the subcellular dynamics of CDPKs and the specificity, as well as the diversity of substrate phosphorylation, are essential for orchestrating various signaling events, allowing a fine tune of the plant innate immune responses.

Presently, a major challenge for studying CDPK functions is to determine and distinguish substrate phosphorylation events responsible for distinct signaling pathways in PTI and ETI. To achieve this goal, a combination of diverse biochemical approaches have been deployed in identifying potential phosphorylation sites in plants. Proteome-based surveys of candidate substrate proteins followed by mass spectrometry, as well as constructing custom-designed peptide libraries, provide a strategy to rapidly test a large number of mapped phosphorylation sites for their potential to be phosphorylated by a specific kinase [94]. Using SRM for example, several serine residues, including Ser-39, Ser-148, Ser-163, and Ser-347, in AtRbohD were identified as the phosphorylation sites of AtCPK5 [77]. Another challenge is to elucidate the convergent points and mechanisms for the crosstalk between MAPK and CDPK signaling pathways, especially how differential signaling components of CDPKs and MAPKs are integrated in different subcellular compartments. Finally, it would be interesting to investigate whether, and how, the CDPK family coordinate with other Ca^2+^-sensor proteins, such as CaMs, CMLs, and CBL/CIPK, in translating the Ca^2+^ signals from the cytosol into the nucleus to subsequently activate the corresponding physiological responses when challenged by pathogens.

## References

[1] Hashimoto K., Kudla J. (2011). Calcium decoding mechanisms in plants. Biochimie.

[2] DeFalco T.A., Bender K.W., Snedden W.A. (2010). Breaking the code: Ca^2+^ sensors in plant signalling. Biochem. J..

[3] Sanders D., Pelloux J., Brownlee C., Harper J.F. (2002). Calcium at the crossroads of signaling. Plant Cell.

[4] Sanders D., Brownlee C., Harper J.F. (1999). Communicating with calcium. Plant Cell.

[5] Harmon A.C., Gribskov M., Harper J.F. (2000). CDPKs-a kinase for every Ca^2+^ signal?. Trends Plant Sci..

[6] Cheng S.H., Willmann M.R., Chen H.C., Sheen J. (2002). Calcium signaling through protein kinases. The *Arabidopsis* calcium-dependent protein kinase gene family. Plant Physiol..

[7] Zielinski R.E. (1998). Calmodulin and calmodulin-binding proteins in plants. Annu. Rev. Plant Physiol. Plant Mol. Biol..

[8] Bouche N., Yellin A., Snedden W.A., Fromm H. (2005). Plant-specific calmodulin-binding proteins. Annu. Rev. Plant Biol..

[9] Luan S., Kudla J., Rodriguez-Concepcion M., Yalovsky S., Gruissem W. (2002). Calmodulins and calcineurin B-like proteins: Calcium sensors for specific signal response coupling in plants. Plant Cell.

[10] Yamniuk A.P., Vogel H.J. (2005). Structural investigation into the differential target enzyme regulation displayed by plant calmodulin isoforms. Biochemistry.

[11] Lee S.H., Johnson J.D., Walsh M.P., van Lierop J.E., Sutherland C., Xu A., Snedden W.A., Kosk-Kosicka D., Fromm H., Naravanan N. (2000). Differential regulation of Ca^2+^/calmodulin-dependent enzymes by plant calmodulin isoforms and free Ca^2+^ concentration. Biochem. J..

[12] Batistic O., Kudla J. (2004). Integration and channeling of calcium signaling through the CBL calcium sensor/CIPK protein kinase network. Planta.

[13] Batistic O., Kudla J. (2012). Analysis of calcium signaling pathways in plants. Biochim. Biophys. Acta.

[14] Golovkin M., Reddy A.S. (2003). A calmodulin-binding protein from *Arabidopsis* has an essential role in pollen germination. Proc. Natl. Acad. Sci. USA.

[15] Vos J.W., Safadi F., Reddy A.S., Hepler P.K. (2000). The kinesin-like calmodulin binding protein is differentially involved in cell division. Plant Cell.

[16] Yamaguchi T., Aharon G.S., Sottosanto J.B., Blumwald E. (2005). Vacuolar Na^+^/H^+^ antiporter cation selectivity is regulated by calmodulin from within the vacuole in a Ca^2+^- and pH-dependent manner. Proc. Natl. Acad. Sci. USA.

[17] Kim M.C., Panstruga R., Elliott C., Muller J., Devoto A., Yoon H.W., Park H.C., Cho M.J., Schulze-Lefert P. (2002). Calmodulin interacts with MLO protein to regulate defence against mildew in barley. Nature.

[18] Ali R., Ma W., Lemtiri-Chlieh F., Tsaltas D., Leng Q., Bodman S., Berkowitz G.A. (2007). Death don’t have no mercy and neither does calcium: *Arabidopsis* CYCLIC NUCLEOTIDE GATED CHANNEL2 and innate immunity. Plant Cell.

[19] Harding S.A., Oh S.H., Roberts D.M. (1997). Transgenic tobacco expressing a foreign calmodulin gene shows an enhanced production of active oxygen species. EMBO J..

[20] Reddy A.S., Ali G.S., Celesnik H., Day I.S. (2011). Coping with stresses: Roles of calcium- and calcium/calmodulin-regulated gene expression. Plant Cell.

[21] Popescu S.C., Popescu G.V., Bachan S., Zhang Z., Seay M., Gerstein M., Snyder M., Dinesh-Kumar S.P. (2007). Differential binding of calmodulin-related proteins to their targets revealed through high-density *Arabidopsis* protein microarrays. Proc. Natl. Acad. Sci. USA.

[22] Wang L., Tsuda K., Sato M., Cohen J.D., Katagiri F., Glazebrook J. (2009). *Arabidopsis* CaM binding protein CBP60g contributes to MAMP-induced SA accumulation and is involved in disease resistance against *Pseudomonas syringae*. PLoS Pathog..

[23] Kolukisaoglu U., Weinl S., Blazevic D., Batistic O., Kudla J. (2004). Calcium sensors and their interacting protein kinases: Genomics of the *Arabidopsis* and rice CBL-CIPK signaling networks. Plant Physiol..

[24] Albrecht V., Ritz O., Linder S., Harter K., Kudla J. (2001). The NAF domain defines a novel protein-protein interaction module conserved in Ca^2+^-regulated kinases. EMBO J..

[25] Boudsocq M., Sheen J. (2013). CDPKs in immune and stress signaling. Trends Plant Sci..

[26] Asai S., Ichikawa T., Nomura H., Kobayashi M., Kamiyoshihara Y., Mori H., Kadota Y., Zipfel C., Jones J.D., Yoshioka H. (2013). The variable domain of a plant Calcium-dependent Protein Kinase (CDPK) confers subcellular localization and substrate recognition for NADPH oxidase. J. Biol. Chem..

[27] Sheen J. (1996). Ca^2+^-dependent protein kinases and stress signal transduction in plants. Science.

[28] Harper J.F., Breton G., Harmon A. (2004). Decoding Ca(2+) signals through plant protein kinases. Annu. Rev. Plant Biol..

[29] Harper J.F., Sussman M.R., Schaller G.E., Putnam-Evans C., Charbonneau H., Harmon A.C. (1991). A calcium-dependent protein kinase with a regulatory domain similar to calmodulin. Science.

[30] Harper J.F., Harmon A. (2005). Plants, symbiosis and parasites: A calcium signalling connection. Nat. Rev. Mol. Cell Biol..

[31] Wernimont A.K., Amani M., Qiu W., Pizarro J.C., Artz J.D., Lin Y.H., Lew J., Hutchinson A., Hui R. (2011). Structures of parasitic CDPK domains point to a common mechanism of activation. Proteins.

[32] Wernimont A.K., Artz J.D., Finerty P., Lin Y.H., Amani M., Allali-Hassani A., Senisterra G., Vedadi M., Tempel W., Mackenzie F. (2010). Structures of apicomplexan calcium-dependent protein kinases reveal mechanism of activation by calcium. Nat. Struct. Mol. Biol..

[33] Jeong J.C., Shin D., Lee J., Kang C.H., Baek D., Cho M.J., Kim M.C., Yun D.J. (2007). Isolation and characterization of a novel calcium/calmodulin-dependent protein kinase, AtCK, from arabidopsis. Mol. Cells.

[34] Asano T., Tanaka N., Yang G., Hayashi N., Komatsu S. (2005). Genome-wide identification of the rice calcium-dependent protein kinase and its closely related kinase gene families: Comprehensive analysis of the CDPKs gene family in rice. Plant Cell Physiol..

[35] Klimecka M., Muszynska G. (2007). Structure and functions of plant calcium-dependent protein kinases. Acta Biochim. Pol..

[36] Myers C., Romanowsky S.M., Barron Y.D., Garg S., Azuse C.L., Curran A., Davis R.M., Hatton J., Harmon A.C., Harper J.F. (2009). Calcium-dependent protein kinases regulate polarized tip growth in pollen tubes. Plant J..

[37] Ivashuta S., Liu J., Liu J., Lohar D.P., Haridas S., Bucciarelli B., VandenBosch K.A., Vance C.P., Harrison M.J., Gantt J.S. (2005). RNA interference identifies a calcium-dependent protein kinase involved in *Medicago truncatula* root development. Plant Cell.

[38] Matschi S., Werner S., Schulze W.X., Legen J., Hilger H.H., Romeis T. (2013). Function of calcium-dependent protein kinase CPK28 of *Arabidopsis thaliana* in plant stem elongation and vascular development. Plant J..

[39] Zhu S.Y., Yu X.C., Wang X.J., Zhao R., Li Y., Fan R.C., Shang Y., Du S.Y., Wang X.F., Wu F.Q. (2007). Two calcium-dependent protein kinases, CPK4 and CPK11, regulate abscisic acid signal transduction in *Arabidopsis*. Plant Cell.

[40] Ye W., Muroyama D., Munemasa S., Nakamura Y., Mori I.C., Murata Y. (2013). Calcium-dependent protein kinase, CPK6, positively functions in induction by YEL of stomatal closure and inhibition by YEL of light-induced stomatal opening in *Arabidopsis*. Plant Physiol..

[41] Munemasa S., Hossain M.A., Nakamura Y., Mori I.C., Murata Y. (2011). The *Arabidopsis* calcium-dependent protein kinase, CPK6, functions as a positive regulator of methyl jasmonate signaling in guard cells. Plant Physiol..

[42] Takeuchi O., Akira S. (2010). Pattern recognition receptors and inflammation. Cell.

[43] Ausubel F.M. (2005). Are innate immune signaling pathways in plants and animals conserved?. Nat. Immunol..

[44] Boller T., Felix G. (2009). A renaissance of elicitors: Perception of microbe-associated molecular patterns and danger signals by pattern-recognition receptors. Annu. Rev. Plant Biol..

[45] Chisholm S.T., Coaker G., Day B., Staskawicz B.J. (2006). Host-microbe interactions: Shaping the evolution of the plant immune response. Cell.

[46] Erbs G., Silipo A., Aslam S., de Castro C., Liparoti V., Flagiello A., Pucci P., Lanzetta R., Parrilli M., Molinaro A. (2008). Peptidoglycan and muropeptides from pathogens *Agrobacterium* and *Xanthomonas* elicit plant innate immunity: Structure and activity. Chem. Biol..

[47] Zipfel C. (2008). Pattern-recognition receptors in plant innate immunity. Curr. Opin. Immunol..

[48] Erbs G., Newman M.A. (2012). The role of lipopolysaccharide and peptidoglycan, two glycosylated bacterial microbe-associated molecular patterns (MAMPs), in plant innate immunity. Mol. Plant Pathol..

[49] Miya A., Albert P., Shinya T., Desaki Y., Ichimura K., Shirasu K., Narusaka Y., Kawakami N., Kaku H., Shibuya N. (2007). CERK1, a LysM receptor kinase, is essential for chitin elicitor signaling in *Arabidopsis*. Proc. Natl. Acad. Sci. USA.

[50] Chinchilla D., Zipfel C., Robatzek S., Kemmerling B., Nurnberger T., Jones J.D.G., Felix G., Boller T. (2007). A flagellin-induced complex of the receptor FLS2 and BAK1 initiates plant defence. Nature.

[51] Zhang J., Zhou J.M. (2010). Plant immunity triggered by microbial molecular signatures. Mol. Plant.

[52] Lu D., Wu S., Gao X., Zhang Y., Shan L., He P. (2010). A receptor-like cytoplasmic kinase, BIK1, associates with a flagellin receptor complex to initiate plant innate immunity. Proc. Natl. Acad. Sci. USA.

[53] Lin W., Lu D., Gao X., Jiang S., Ma X., Wang Z., Mengiste T., He P., Shan L. (2013). Inverse modulation of plant immune and brassinosteroid signaling pathways by the receptor-like cytoplasmic kinase BIK1. Proc. Natl. Acad. Sci. USA.

[54] Lu D., Lin W., Gao X., Wu S., Cheng C., Avila J., Heese A., Devarenne T.P., He P., Shan L. (2011). Direct ubiquitination of pattern recognition receptor FLS2 attenuates plant innate immunity. Science.

[55] Eitas T.K., Dangl J.L. (2010). NB-LRR proteins: Pairs, pieces, perception, partners, and pathways. Curr. Opin. Plant Biol..

[56] Elmore J.M., Lin Z.J., Coaker G. (2011). Plant NB-LRR signaling: Upstreams and downstreams. Curr. Opin. Plant Biol..

[57] DeYoung B.J., Innes R.W. (2006). Plant NBS-LRR proteins in pathogen sensing and host defense. Nat. Immunol..

[58] Davis B.K., Wen H., Ting J.P. (2011). The inflammasome NLRs in immunity, inflammation, and associated diseases. Annu. Rev. Immunol..

[59] Dodds P.N., Rathjen J.P. (2010). Plant immunity: Towards an integrated view of plant-pathogen interactions. Nat. Rev. Genet..

[60] Boller T., He S.Y. (2009). Innate immunity in plants: An arms race between pattern recognition receptors in plants and effectors in microbial pathogens. Science.

[61] Jones J.D., Dangl J.L. (2006). The plant immune system. Nature.

[62] Gawehns F., Cornelissen B.J., Takken F.L. (2013). The potential of effector-target genes in breeding for plant innate immunity. Microb. Biotechnol..

[63] Dangl J.L., Horvath D.M., Staskawicz B.J. (2013). Pivoting the plant immune system from dissection to deployment. Science.

[64] Nimchuk Z., Eulgem T., Holt B.F., Dangl J.L. (2003). Recognition and response in the plant immune system. Annu. Rev. Genet..

[65] Kunkel B.N., Bent A.F., Dahlbeck D., Innes R.W., Staskawicz B.J. (1993). RPS2, an *Arabidopsis* disease resistance locus specifying recognition of *Pseudomonas syringae* strains expressing the avirulence gene avrRpt2. Plant Cell.

[66] Mindrinos M., Katagiri F., Yu G.L., Ausubel F.M. (1994). The *A. thaliana* disease resistance gene RPS2 encodes a protein containing a nucleotide-binding site and leucine-rich repeats. Cell.

[67] Grant M.R., Godiard L., Straube E., Ashfield T., Lewald J., Sattler A., Innes R.W., Dangl J.L. (1995). Structure of the *Arabidopsis* RPM1 gene enabling dual specificity disease resistance. Science.

[68] Axtell M.J., Staskawicz B.J. (2003). Initiation of RPS2-specified disease resistance in *Arabidopsis* is coupled to the AvrRpt2-directed elimination of RIN4. Cell.

[69] Gao Z., Chung E.H., Eitas TK., Dangl J.L. (2011). Plant intracellular innate immune receptor Resistance to *Pseudomonas syringae* pv. *maculicola* 1 (RPM1) is activated at, and functions on, the plasma membrane. Proc. Natl. Acad. Sci. USA.

[70] Ranf S., Eschen-Lippold L., Pecher P., Lee J., Scheel D. (2011). Interplay between calcium signalling and early signalling elements during defence responses to microbe- or damage-associated molecular patterns. Plant J..

[71] Grant M., Brown I., Adams S., Knight M., Ainslie A., Mansfield J. (2000). The RPM1 plant disease resistance gene facilitates a rapid and sustained increase in cytosolic calcium that is necessary for the oxidative burst and hypersensitive cell death. Plant J..

[72] Geng S., Li A., Tang L., Yin L., Wu L., Lei C., Guo X., Zhang X., Jiang G., Zhai W. (2013). TaCPK2-A, a calcium-dependent protein kinase gene that is required for wheat powdery mildew resistance enhances bacterial blight resistance in transgenic rice. J. Exp. Bot..

[73] Coca M., San Segundo B. (2010). AtCPK1 calcium-dependent protein kinase mediates pathogen resistance in *Arabidopsis*. Plant J..

[74] Boudsocq M., Willmann M.R., McCormack M., Lee H., Shan L., He P., Bush J., Cheng S., Sheen J. (2010). Differential innate immune signalling via Ca(2+) sensor protein kinases. Nature.

[75] Ludwig A.A., Saitoh H., Felix G., Freymark G., Miersch O., Wasternack C., Boller T., Jones J.D., Romeis T. (2005). Ethylene-mediated cross-talk between calcium-dependent protein kinase and MAPK signaling controls stress responses in plants. Proc. Natl. Acad. Sci. USA.

[76] Kobayashi M., Ohura I., Kawakita K., Yokota N., Fujiwara M., Shimamoto K., Doke N., Yoshioka H. (2007). Calcium-dependent protein kinases regulate the production of reactive oxygen species by potato NADPH oxidase. Plant Cell.

[77] Dubiella U., Seybold H., Durian G., Komander E., Lassig R., Witte C.P., Schulze W.X., Romeis T. (2013). Calcium-dependent protein kinase/NADPH oxidase activation circuit is required for rapid defense signal propagation. Proc. Natl. Acad. Sci. USA.

[78] Asano T., Hayashi N., Kobayashi M., Aoki N., Miyao A., Mitsuhara I., Ichikawa H., Komatsu S., Hirochika H., Kikuchi S. (2012). A rice calcium-dependent protein kinase OsCPK12 oppositely modulates salt-stress tolerance and blast disease resistance. Plant J..

[79] Liese A., Romeis T. (2013). Biochemical regulation of *in vivo* function of plant calcium-dependent protein kinases (CDPK). Biochim. Biophys. Acta.

[80] Lee J., Rudd J.J., Macioszek V.K., Scheel D. (2004). Dynamic changes in the localization of MAPK cascade components controlling pathogenesis-related (PR) gene expression during innate immunity in parsley. J. Biol. Chem..

[81] Wurzinger B., Mair A., Pfister B., Teige M. (2011). Cross-talk of calcium-dependent protein kinase and MAP kinase signaling. Plant Signal. Behav..

[82] Gao X., Chen X., Lin W., Chen S., Lu D., Niu Y., Li L., Cheng C., McCormack M., Sheen J. (2013). Bifurcation of *Arabidopsis* NLR immune signaling via Ca^2+^-dependent protein kinases. PLoS Pathog..

[83] Gust A.A., Biswas R., Lenz H.D., Rauhut T., Ranf S., Kemmerling B., Gotz F., Glawischnig E., Lee J., Felix G. (2007). Bacteria-derived peptidoglycans constitute pathogen-associated molecular patterns triggering innate immunity in *Arabidopsis*. J. Biol. Chem..

[84] Blume B., Nurnberger T., Nass N., Scheel D. (2000). Receptor-mediated increase in cytoplasmic free calcium required for activation of pathogen defense in parsley. Plant Cell.

[85] Zimmermann S., Nurnberger T., Frachisse J.M., Wirtz W., Guern J., Hedrich R., Scheel D. (1997). Receptor-mediated activation of a plant Ca^2+^-permeable ion channel involved in pathogen defense. Proc. Natl. Acad. Sci. USA.

[86] Ma W., Smigel A., Tsai Y.C., Braam J., Berkowitz G.A. (2008). Innate immunity signaling: Cytosolic Ca^2+^ elevation is linked to downstream nitric oxide generation through the action of calmodulin or a calmodulin-like protein. Plant Physiol..

[87] Romeis T., Ludwig A.A., Martin R., Jones J.D. (2001). Calcium-dependent protein kinases play an essential role in a plant defence response. EMBO J..

[88] Freymark G., Diehl T., Miklis M., Romeis T., Panstruga R. (2007). Antagonistic control of powdery mildew host cell entry by barley calcium-dependent protein kinases (CDPKs). Mol. Plant Microbe Interact..

[89] Martin M.L., Busconi L. (2000). Membrane localization of a rice calcium-dependent protein kinase (CDPK) is mediated by myristoylation and palmitoylation. Plant J..

[90] Chehab E.W., Patharkar O.R., Hegeman A.D., Taybi T., Cushman J.C. (2004). Autophosphorylation and subcellular localization dynamics of a salt- and water deficit-induced calcium-dependent protein kinase from ice plant. Plant Physiol..

[91] Patharkar O.R., Cushman J.C. (2000). A stress-induced calcium-dependent protein kinase from *Mesembryanthemum crystallinum* phosphorylates a two-component pseudo-response regulator. Plant J..

[92] Messinese E., Mun J.H., Yeun L.H., Jayaraman D., Rouge P., Barre A., Lougnon G., Schornack S., Bono J.J., Cook D.R. (2007). A novel nuclear protein interacts with the symbiotic DMI3 calcium- and calmodulin-dependent protein kinase of *Medicago truncatula*. Mol. Plant Microbe Interact.

[93] Charpentier M., Oldroyd G.E. (2013). Nuclear calcium signaling in plants. Plant Physiol..

[94] Curran A., Chang I.F., Chang C.L., Garg S., Miguel R.M., Barron Y.D., Li Y., Romanowsky S., Cushman J.C., Gribskov M. (2011). Calcium-dependent protein kinases from *Arabidopsis* show substrate specificity differences in an analysis of 103 substrates. Front Plant Sci..

[95] Shen Q.H., Saijo Y., Mauch S., Biskup C., Bieri S., Keller B., Seki H., Ulker B., Somssich I.E., Schulze-Lefert P. (2007). Nuclear activity of MLA immune receptors links isolate-specific and basal disease-resistance responses. Science.

[96] Bhattacharjee S., Halane M.K., Kim S.H., Gassmann W. (2011). Pathogen effectors target Arabidopsis EDS1 and alter its interactions with immune regulators. Science.

[97] Heidrich K., Wirthmueller L., Tasset C., Pouzet C., Deslandes L., Parker J.E. (2011). Arabidopsis EDS1 connects pathogen effector recognition to cell compartment-specific immune responses. Science.

[98] Gao X., He P. (2013). Nuclear dynamics of Arabidopsis calcium-dependent protein kinases in effector-triggered immunity. Plant Signal. Behav..

